# *AFAP1‐AS1* is upregulated and promotes esophageal squamous cell carcinoma cell proliferation and inhibits cell apoptosis

**DOI:** 10.1002/cam4.848

**Published:** 2016-08-30

**Authors:** Hong‐lei Luo, Ming‐de Huang, Jia‐ni Guo, Rui‐hua Fan, Xiao‐tian Xia, Jing‐dong He, Xiao‐fei Chen

**Affiliations:** ^1^ Department of RadiotherapyHuai'an First People's HospitalNanjing Medical UniversityHuai'anJiangsu223300China; ^2^ Department of Medical OncologyHuai'an First People's HospitalNanjing Medical UniversityHuai'anJiangsu223300China

**Keywords:** *AFAP1‐AS1*, esophageal squamous cell carcinoma, malignant proliferation, noncoding RNA

## Abstract

Recent findings indicate that long noncoding RNAs (lncRNAs) were dysregulated in many kinds of tumors including esophageal squamous cell carcinoma (ESCC). LncRNA *AFAP1‐AS1* was found to be upregulated in hepatocellular carcinoma (HCC), lung cancer, colorectal cancer, esophageal adenocarcinoma (EAC), pancreatic ductal adenocarcinoma, and nasopharyngeal carcinoma, while its clinical value and potential function in ESCC are still unknown. Expression of *AFAP1‐AS1* was measured in 65 ESCC tissues and corresponding noncancerous tissues by quantitative real‐time polymerase chain reaction, which revealed that *AFAP1‐AS1* expression was markedly elevated in ESCC tissues and significantly associated with advanced TNM stage (*P* = 0.004) and larger tumor size (*P* = 0.040). Moreover, by knocking down *AFAP1‐AS1* expression in ESCC cells, the proliferation and colony‐forming ability were inhibited and cell apoptosis was induced. Our data indicated the first time that *AFAP1‐AS1*, a novel oncogene, was remarkably upregulated and played a critical role in the progression of ESCC.

## Introduction

Esophageal squamous cell carcinoma (ESCC) is one of the main causes of cancer death in China 1. Owing to its increasing incidence and worse prognosis, more and more researchers are committed to the early detection of ESCC. Recently, increasing evidence has demonstrated that long noncoding RNAs (lncRNAs), comprise noncoding RNAs longer than 200 nucleotides in length, are pervasively transcribed in the genome and may participate in the regulation of cellular processes as crucial factors, such as cellular differentiation, proliferation, cell cycle regulation, and metastasis 2. Simultaneously, many lncRNAs are demonstrated to be differentially expressed in a series of cancers, such as MALAT‐1 in lung cancer 3, HULC in hepatocellular carcinoma 4, and HOTAIR in pancreatic cancer 5. In addition, numerous evidence indicated that lncRNAs could take part in a broad range of signal pathways and act as either oncogene or tumor suppressor gene depending on their targets. However, to the best of our knowledge, studies of lncRNAs in ESCC were seldom reported.

*AFAP1‐AS1*, which is derived from the antisense strand at the AFAP1 coding gene locus, has been reported to be upregulated in esophageal adenocarcinoma (EAC) tissues and cell lines 6. Moreover, inhibition of its expression in EAC cells resulted in diminished cell growth, migration, invasion, along with increased apoptosis. On the basis of previous study, our current research focuses on the role of *AFAP1‐AS1* in ESCC. We found that the expression of *AFAP1‐AS1* was dramatically upregulated in ESCC tissues and cell lines. Further functional studies revealed that knockdown of *AFAP1‐AS1* expression in ESCC cells could result in diminished cell growth and increased apoptosis, which suggested that *AFAP1‐AS1* was a potential oncogene of ESCC.

## Materials and Methods

### Patients and tissue specimens

A total of 65 patients, who underwent radical surgery for ESCC at Huai'an First People's Hospital, Nanjing Medical University (Huai'an, China), were selected to participate in this study. Both ESCC and corresponding adjacent specimens were collected before adjunctive therapy and the diagnosis of ESCC was confirmed by histopathology. Data of all patients including age, gender, history of smoking and drinking, ESCC tumor size, and pTNM stage were obtained from clinical material and pathology reports in 2012. After surgical resection of ESCC, the specimens were immediately collected and frozen at −80°C.

### Ethical approval of the study protocol

This research was in accordance with the requirements of the Declaration of Helsinki. Written informed consent was received from the ESCC patients before specimen collection. Our study followed the institutional ethical guidelines approved by Huai'an First People's Hospital, Nanjing Medical University (Huai'an, China).

### Cell culture

Two esophageal carcinoma cell lines (ECA‐109 and TE‐1) were purchased from Shanghai Institutes for Biological Sciences (Shanghai, China), while a normal human esophageal epithelial cell line (HEEC) was obtained from ScienCell Research Laboratories (Carlsbad, CA 92011, USA). All the cell lines were maintained according to the vendor's instructions. ECA‐109 and TE‐1 cells were maintained in Dulbecco's modified Eagle's medium (DMEM; GIBCO‐BRL, USA) and HEEC cells were cultured in RPMI 1640 medium (GIBCO‐BRL, USA), supplemented with 10% FBS, 100U/mL penicillin sodium, and 100 mg/mL streptomycin sulfate. All cells were cultured in a 37°C incubator containing 5% CO_2_. The morphological changes of cells were observed daily under the inverted microscope. The medium were replaced every 3 days to discard the cells which were not adherent.

### Cell transfection

After reaching more than 50% confluence, the ESCC cell lines were transfected with specific siRNA oligonucleotides. Three different siRNAs were designed to ensure transfection efficiency and avoid off‐target effects. After verification, two siRNAs were thought to be appropriate for *AFAP1‐AS1* knockdown (Fig. 2B) (Invitrogen, Grand Island, NY, USA). Negative control siRNA (si‐NC) was purchased from Invitrogen at the same time. Cells were seeded at 6‐well plates for 24 h and then transfected with designed siRNA (100 nmol/L) and si‐NC (100 nmol/L), respectively, by Lipofectamine RNAi MAX in serum‐free medium, according to the manufacturer's protocols (Invitrogen, Grand Island, NY, USA). Cells, after transfection, were harvested for following analyses. The sequences of the AFAP1‐AS1 targeting siRNAs are summarized in Table 1.

**Table 1:** The sequence for primers and siRNA

Primers used for qRT‐PCR	
GAPDH F	GGGAGCCAAAAGGGTCAT
GAPDH R	GAGTCCTTCCACGATACCAA
AFAP1‐AS1 F	AGCCTGTTGAATCAGCCAACT
AFAP1‐AS1 R	GGTTCATACCAGCCCTGTCC
siRNAs oligonucleotides	
si‐AFAP1‐AS1‐1#	AUUUGAUGCCAGUUCAGUAGAGCCG
si‐AFAP1‐AS1‐2#	GCCAUGUCAUCUGACUGGCUCUGAA
si‐AFAP1‐AS1‐3#	CAACACCUGCCUUCCCUCCUCUAAA

### RNA isolation and qRT‐PCR

Total RNA was isolated from tissues or cultured cells treated with Trizol reagent (Life Technologies, Carlsbad, CA, USA). One microgram of total RNA was used for the reverse transcription reaction in a final volume of 20 *μ*L with random primers under standard conditions using PrimeScript RT Reagent Kit with gDNA Eraser (Takara, Dalian, China). 1 *μ*L of the corresponding cDNA was used for subsequent qRT‐PCR reactions using SYBR Premix Ex Taq (Takara, Dalian, China) according to the manufacturer's instructions. The expression of GAPDH was used to normalize the results. The PCR amplification was performed for 40 cycles of 95°C for 5 sec, 60°C for 34 sec, and 68°C for 20 sec on an ABI 7500 Real‐Time PCR System (Applied Biosystems, Foster City, CA). All reactions were run in triplicate and data were analyzed using the comparative cycle threshold (CT) method. The primer sequences are summarized in Table 1.

### Cell proliferation assays

For the cell proliferation assay, a density of 3000 cells per well was seeded in 96‐well plates at day 0 (24 h after siRNA transfection). Cell proliferation was determined at 24, 48, 72, and 96 h and measured by Cell Proliferation Reagent Kit I (MTT) (Roche, Basel, Switzerland). For the colony formation assay, 500 transfected cells were plated into a 6‐well plate and incubated in DMEM containing 10% FBS, being replaced every 4 days. Two weeks later, colonies fixed in methanol were stained with 0.1% crystal violet (Sigma‐Aldrich, St. Louis, MO) for 15 min. The colony formation was then manually counted. Clones containing more than 50 cells were counted using a grid. Three independent experiments were carried out. The formula for the colony formation ratio was as follows: Ratio = Numbers of Colony/Initiative Cells × 100%.

### Apoptosis assay

ECA109 or TE‐1 cells for cell apoptosis analysis were collected 48 h after transfection with si‐*AFAP1‐AS1* or negative control. After staining with FITC‐Annexin V and PI, the apoptosis assay was performed using the FITC‐Annexin V Apoptosis Detection Kit (BD Biosciences, San Jose, CA, USA) according to the manufacturer's recommendations. Cells were then analyzed with a FACScan flow cytometry system (BD Biosciences, San Jose, CA, USA) equipped with Cell Quest software (BD Biosciences, San Jose, CA, USA). The relative ratio of early apoptotic cells and late apoptotic cells were compared to negative control transfectant, respectively.

### Statistical analysis

The SPSS 17.0 software (IBM, Chicago, IL) was used to determine statistical difference in each experiment. The result was expressed as mean ± SD. Significance between groups was tested using paired Student's t test, Wilcoxon test or Pearson's chi‐squared test. *P* < 0.05 was considered to be statistically significant.

## Results

### *AFAP1‐AS1* is upregulated in ESCC tissues and correlated with tumor size and TNM stage

**Figure 1:**
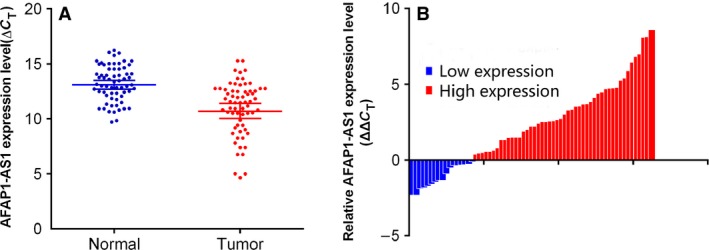
Relative AFAP1‐AS1 expression in esophageal squamous cell carcinoma (ESCC) tissues (A, B). Relative AFAP1‐AS1 expression in ESCC tissues (*n* = 65) compared with corresponding nontumor tissues (*n* = 65). AFAP1‐AS1 expression was examined by qPCR and normalized to GAPDH expression. Results were presented as Δ cycle threshold (CT) in tumor tissues relative to normal tissues. (B) Relative AFAP1‐AS1 expression in ESCC tissues (*n* = 65) compared with corresponding nontumor tissues (*n* = 65). AFAP1‐AS1 expression was classified into two groups. Positive ΔΔCT meant high AFAP1‐AS1 expression. Negative ΔΔCT meant low AFAP1‐AS1 expression.

The expression of AFAP1‐AS1 in 65 paired ESCC tissues and corresponding adjacent tissues was observed by qRT‐PCR, which showed that AFAP1‐AS1 expression in ESCC was significantly elevated in 73.84%(48 of 65, fold ≧1.0) (*P* < 0.01) (Fig. 1A and B). Moreover, we evaluated the potential correlation between *AFAP1‐AS1* expression and patients' clinical features that are shown in Table 2. Importantly, high expression of *AFAP1‐AS1* in ESCC was associated with tumor size (*P *= 0.040) and advanced TNM stage (*P *= 0.004). However, other parameters, such as gender (*P *= 0.451), age (*P *= 0.449), drinking status (*P *= 0.508), and smoking status (*P *= 0.880) were not associated with *AFAP1‐AS1* in ESCC.

**Table 2:** Correlation between AFAP1‐AS1 expression and clinicopathological characteristics in esophageal squamous cell carcinoma

Clinical parameter	AFAP1‐AS1		Chi‐squared test P value
	High no. cases	Low no. cases	
Age (years)			0.449
≤64	25	12	
>64	25	8	
Gender			0.451
Male	37	13	
Female	13	7	
Smoking state			0.880
Yes	24	10	
No	26	10	
Drinking state			0.508
Yes	41	15	
No	9	5	
Tumor size			0.040
≤3 cm	14	12	
3–5 cm	29	7	
>5 cm	7	1	
Tumor stage			<0.01
I	2	11	
II	27	4	
III	21	5	

### *AFAP1‐AS1* promotes ESCC cells proliferation in vitro

**Figure 2:**
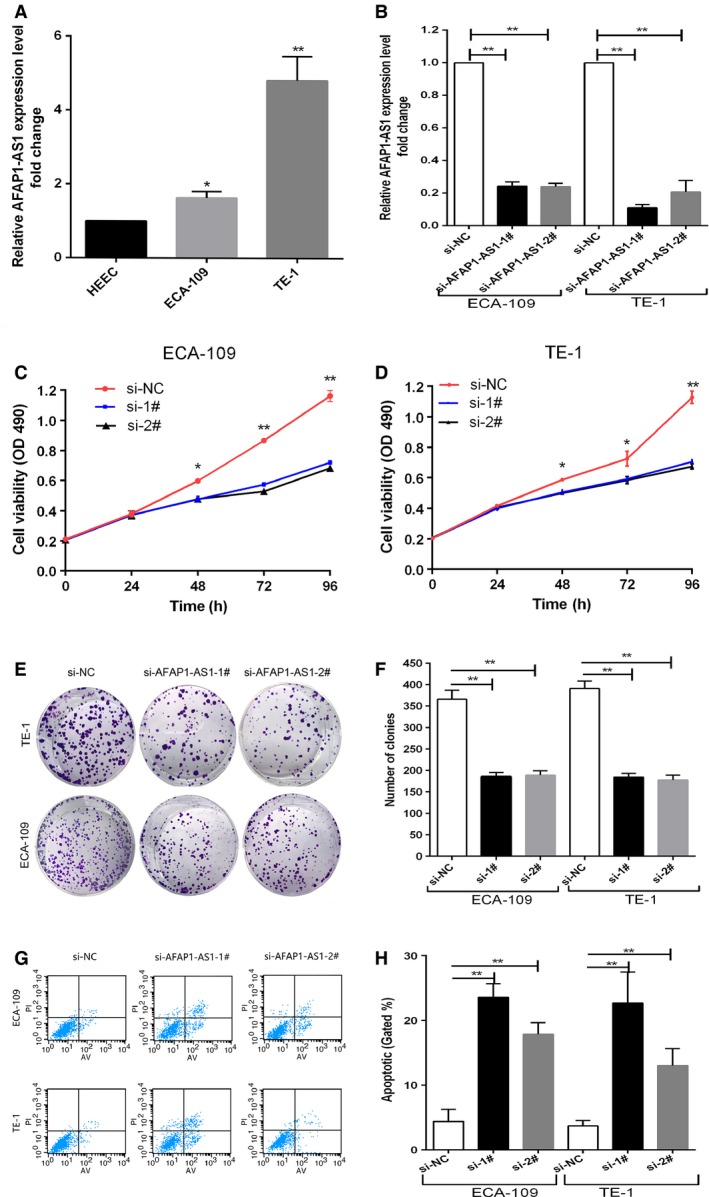
Effects of AFAP1‐AS1 knockdown on viability and apoptosis of esophageal squamous cell carcinoma (ESCC) cells in vitro. (A) Relative AFAP1‐AS1 expression levels of ESCC cell lines (ECA‐109, TE‐1) compared with that in the normal esophageal epithelium cell line(HEEC). (B) The AFAP1‐AS1 expression level was determined by qPCR when ECA‐109 and TE‐1 cells transfected with si‐AFAP1‐AS1. (C, D) MTT assays were used to determine the cell viability for si‐AFAP1‐AS1‐transfected ECA‐109 and TE‐1 cells. Values represented the mean ± SD from three independent experiments. (E, F) Colony‐forming assays were conducted to determine the proliferation of si‐AFAP1‐AS1‐transfected ECA‐109 and TE‐1 cells. (G, H) Flow cytometry assays were performed to analyze the cell apoptosis when ESCC cells were transfected with si‐AFAP1‐AS1 48 h later. *P < 0.05, **P < 0.01.

To investigate whether *AFAP1‐AS1* has an effect on the proliferation of ESCC cells, we examined *AFAP1‐AS1* expression level in two ESCC cell lines (ECA109, TE‐1) and one normal cell line (HEEC). As shown in Figure 2A, the expression levels of *AFAP1‐AS1* in ECA109 and TE‐1 cells were higher than HEEC cells. Afterward, chemically synthesized siRNAs were used to knockdown *AFAP1‐AS1* expression in ECA109/TE1 cells, respectively (Fig. 2B). MTT assay was used to measure cell proliferative activity, which showed that the growth and proliferation of ESCC cells transiently transfected with siRNA1#, and siRNA2# were significantly inhibited compared with negative control groups (Fig. 2C and D). Colony formation assay also demonstrated that the clonogenic survival rate of ECA109 and TE1 cells with *AFAP1‐AS1* knockdown was significantly decreased (Fig. 2E and F).

### Effect of *AFAP1‐AS1* on ESCC cell apoptosis

In addition, to further examine the effect of *AFAP1‐AS1* on cell apoptosis, flow cytometric analysis was performed. The results showed that cell apoptosis was obviously induced after interfering *AFAP1‐AS1* expression in ESCC cells (Fig. 2G and H).

## Discussion

LncRNAs are usually defined as transcribed noncoding RNAs longer than 200 nucleotides 7, 8. Emerging evidence reveal that lncRNAs may play a significant role in multiple physiological and pathological processes, including malignant diseases 9, 10, 11. A lot of new lncRNAs are proved to play critical roles in tumor formation and progression 11.

ESCC is one of the world's most deadly cancers 1. In China, ESCC is the major subtype of esophageal cancer, accounting for over 90% of the cases 12. As a tumor including multiple biological processes, ESCC begins with a variety of genetic and epigenetic changes. Earlier studies have shown that lncRNAs were critical promoters in the tumorigenesis and progression of ESCC, such as SPRY4‐IT1^13^, MALAT1^14^, and CCAT2^15^. Wang et al. 16 recently found that the expression of lncRNA MALAT1 were higher in ESCC tissues and cell lines. Downregulation of MALAT1 decreased the expression of *β*‐catenin, Lin28, and EZH2 genes, while overexpressed EZH2 could reverse such suppressive effect, indicating the promotion mechanism of MALAT1 in ESCC. Other researchers also discovered that several lncRNAs could serve as tumor suppressor genes in ESCC, such as UCA1^17^ and LET 18. Previous studies have demonstrated that AFAP1‐AS1 was dysregulated in a variety of cancers and associated with tumor progression, including EAC 6, hepatocellular carcinoma (HCC) 19, colorectal cancer 20, pancreatic ductal adenocarcinoma 21, lung cancer 22, and nasopharyngeal carcinoma 23. In HCC, AFAP1‐AS1 was proved to be highly expressed and promoted cell proliferation and invasion via upregulation of RhoA/Rac2 signaling 24. Zeng et al.^22^ also found that AFAP1‐AS1 was associated with poor prognosis and promoted cell invasion and metastasis through regulation of actin filament integrity in lung cancer. Especially, AFAP1‐AS1 was found extremely hypomethylated and overexpressed in Barrett's esophagus and esophageal adenocarcinoma, which could inhibit the biologic functions of esophageal adenocarcinoma cells 6. There has been research indicating that AFAP1‐AS1 was upregulated in ESCC and predicted chemoradioresistance and poor prognosis in patients who received definitive chemoradiotherapy 25. However, the exact function of AFAP1‐AS1 in ESCC cell biology remains unknown. Here, we evaluated the potential correlations between AFAP1‐AS1 expression and patients' clinical features. The results showed that there was a significant relationship between high AFAP1‐AS1 expression and tumor size, as well as advanced TNM stage. Consistent with Wu W's research in esophageal adenocarcinoma, our experiment found that AFAP1‐AS1 could promote ESCC cells' proliferation in vitro. Furthermore, the expression of AFAP1‐AS1 was measured in the nuclear and cytosolic fractions, which indicated that AFAP1‐AS1 was mostly distributed in the nucleus, prompting its function in epigenetic or transcriptional regulation, such as histone modification, chromatin remodeling, and regulating target genes or transcription factors. It was previously reported in human non‐small‐cell lung cancer that AFAP1‐AS1 can affect cell proliferation partly through epigenetically regulating the expression of homeobox B7 (HOXB7). Very likely, AFAP1‐AS1 can modulate HOXB7 expression to regulate the proliferation of ESCC cells, which still needs further validation. There is also the possibility that AFAP1‐AS1 can exert its function in ESCC through binding to PRC2, considering that PRC2‐related lncRNAs are involved in multiple cancers. However, the specific molecular mechanism of AFAP1‐AS1 in ESCC remains to be further studied.

## Conflict of Interest

The authors declare that they have no conflict of interests.
